# The work of Arthur Bispo Do Rosário

**DOI:** 10.1017/S204579602000013X

**Published:** 2020-01-29

**Authors:** Coline De Reymaeker

**Affiliations:** Art et marges museum, Outsider art, Brussels, Belgium

**Keywords:** Art Brut, contemporary art, outsider art, self-taught

The work of Arthur Bispo do Rosário propels us into another world, that of the revisiting and subjective reinterpretation of the environment in which the artist lived: from the fork to the boat, the entire personal story and the whole framework of his daily life are to be found therein, or nearly so. To understand the relentless re-creation of his world, it is necessary to trace the peculiar path of this Brazilian artist.

The only law that Bispo respects, and for which he works day and night, is that imposed on him by God and his angels. Rio de Janeiro, midnight, 22 December 1938, Bispo looks at the sky and sees God come down surrounded by seven blue angels: in the throes of delirium and following his irrepressible impulses, Bispo follows a precise path in town, guided by the images of his revelation and by the secrets confided in him at the time. He marched for 2 days following a strange and incongruous itinerary which he would later embroider on a banner in great detail. Bispo cannot but roam the streets of Rio and tell one and all, especially the different clergymen of the city, about his new status, that of God's messenger on earth. But he was never received as he hoped, and his ‘mad’ roaming would ultimately end in the Colônia Juliano Moreira psychiatric hospital (Silva, 1998). There he would embark on – and continue for 50 years until his death in 1989 – his mission of representing the world that the angels entrusted him during a second revelation that occurred in 1939: Bispo has to offer God a representation of the world for the day of his passing into the beyond.

Once institutionalised, he stamped his cell with his own idiosyncrasy and turned it into a sort of temple dedicated to the reconstruction of the universe, a task to which he devoted himself body and soul. By getting personally and totally involved in the representation of the world he is tasked to bring about, he can spend several days working on this re-creation, holed up in his cell, hardly eating anything and cut-off from the surrounding asylum world. One of the frequent signals of his *transformation into a creator* was the question: ‘*Don't you see anything at the top of my head?*’ (Hidalgo, 1996). He claimed that there was a god there who wanted to talk to him. When he is in such a state of delirium, Bispo asks himself to be locked up, predicting that he will be turned into a king and ‘go to war’. Once alone in the heart of his universe, he enriches, transforms and perfects it. He has no contact with the outside anymore, except during his meals, which he refuses most of the time, arguing that he is going to ‘*dry up in order to become a saint*’ or ‘*transparent*’. These periods of intense activity would at times last for whole months during which he would eat only some fruit that the hospital staff brought for him.

At the outset, using only blue thread unravelled from his inmate's uniform and old sheets, Bispo embroidered images and words recounting the highlights of his existence. He used the same blue thread to ‘wrap’ or ‘mummify’ hundreds of objects. He then accumulated shoes, boots, cups, bottles filled with confetti, scrap metal, religious objects, etc., on wooden and cardboard racks, grouped with extraordinary formal rigour – his *showcases.* In addition to *showcases* consisting of day-to-day objects, Bispo also collected thousands of small brown or blue cards similar to identity cards or bibliographical references. Arranged in alphabetical order for the most part, this index classifies and arranges the particulars of everything and everyone.

He also embroidered sashes and sceptres of beauty contestants, made of discarded and enigmatic objects, assembled metal reliefs, organised compositions from plastic packaging or pieces of cloth, made small wooden boats, etc. Thereby portraying the universe in order to accomplish his mission and present the universe (as he had reconstructed it) to God on the day he would meet his maker. In preparation for that long-awaited day, he created with painstaking research the imposing *Manto da apresentação* (Presentation Mantle), a summation of his work in terms of form and meaning: for Bispo, donning this mantle means that he accepts to ‘in-vest himself’ as a guardian of the universe at the behest of God.

At first sight, Bispo's universe is chaotic and unwieldy, but this chaos is only apparent. In Bispo's art, everything is organised with rigour. The embroidered, written or painted registers are numbered and coloured to make them identifiable and classifiable, they give clear instructions, comprise precise descriptions and refer to each other. Precisely dated events are transcribed therein, the index cards are classified in alphabetical order, the panels are carefully prepared and composed to bring together various constituent elements according to consistent criteria like shape, colour, material and utility. Scattered fragments of reality are united by a link (Burrowes, 1999).

**Fig. 1.**
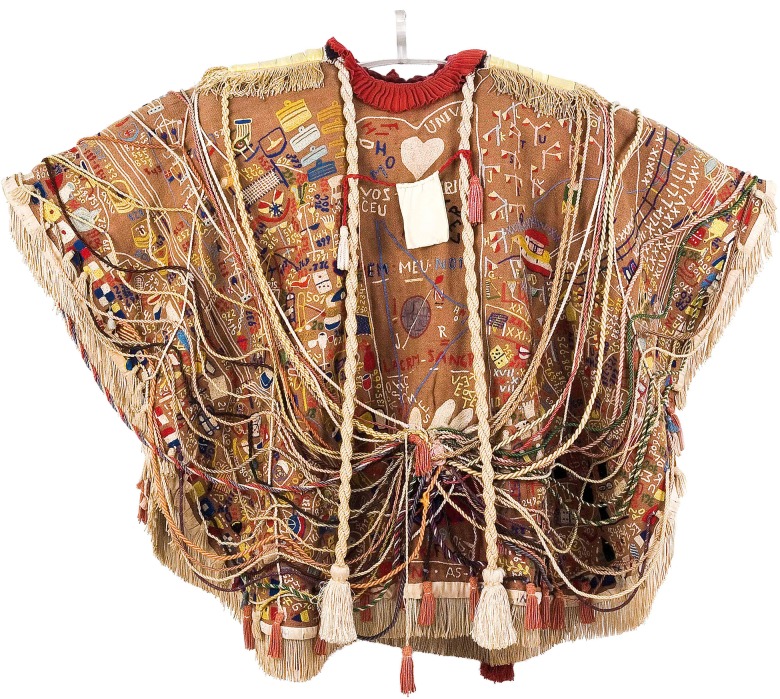
Manto da apresentação - coleção Museu Bispo do Rosário Arte Contemporânea/PCdoRJ ©Rodrigo Lopes.

The inventory of the world presented by Bispo at the end of his life aroused the curiosity of certain stakeholders in Carioca cultural circles. This led to a first exhibition in 1982, when Bispo was still alive, in the Rio Museum of Modern Art. As of 1989, the pace of exhibitions that featured pieces by Bispo picked up, in Brazil and abroad. For instance, he represented Brazil at the 46^th^ Biennale in Venice in 1995 (Clair, 1995).

Approaching these creations calls for a capacity of humility, a recognition of our limits when faced with something that is difficult to grasp, fascinating though it is. Reducing these objects, whether to outsider art or ‘primitive’ art, for instance, to simply aesthetic forms, is to deprive them of the very essence of their particularity, that which nurtures and brings them into being. Accepting not being able to get to the crux of the matter, to know it, is part of the adventure, of the encounter with Bispo's world. An exhaustive comprehension of works proves impossible for as long as they inevitably refer to the world of the artist. And it is precisely this elusive nature that confers on Bispo's art its constant intensity.

**Fig. 2.**
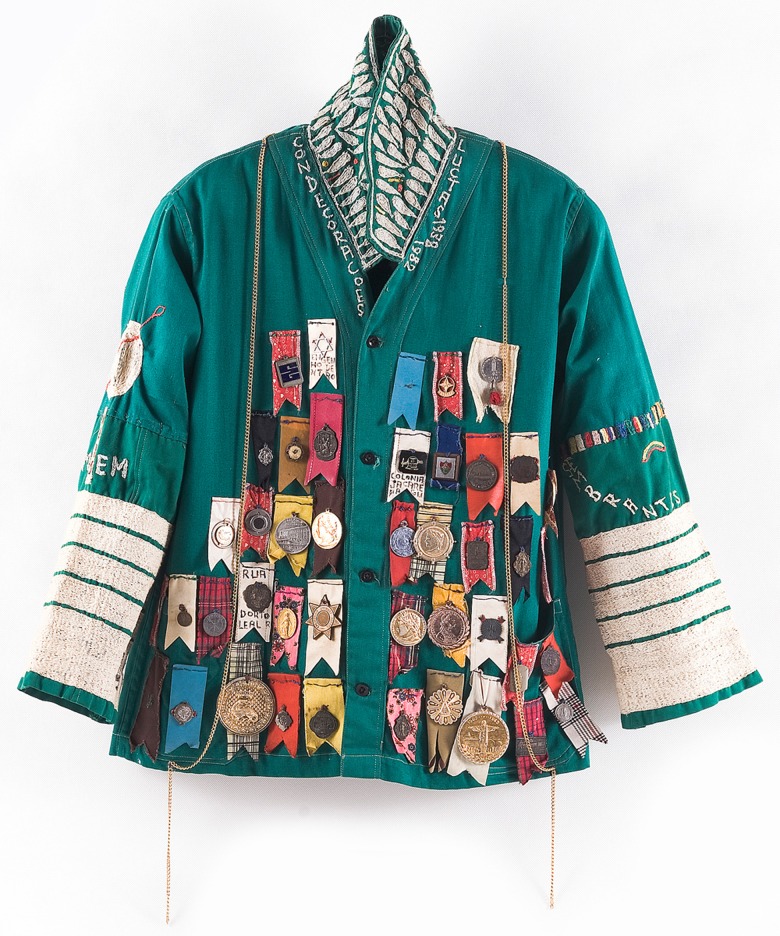
Lutas - coleção Museu Bispo do Rosário Arte Contemporânea/PCdoRJ ©Rodrigo Lopes.

## About the author

Coline De Reymaeker is an art historian and curator specialised in Art Brut. In 2004, she discussed her dissertation on Bispo do Rosario at the Free University of Brussels, and in 2011, during the International Art Festival Europalia Brazil, she presented and co-curated an exhibition on Bispo do Rosario's work at the Art et marges Museum in Brussels. She curated many exhibitions, and in 2018–2019, she was the Director of the Art et marges Museum of Brussels.

Carole Tansella, *Section Editor*

## References

[ref1] Burrowes P (1999) O universo segundo Arthur Bispo do Rosário. Rio de Janeiro: Editora FGV.

[ref2] Clair J (1995) La Biennale di Venezia 46° Esposizione Internazionale d'Arte. Identità Alterità Figure del Corpo 1895/1995. Venezia: Marsilio.

[ref3] De Reymaeker C (2011) Arthur Bispo do Rosário. Exhibition catalogue, Bruxelles: Art et marges Museum.

[ref4] Hidalgo L (1996) Arthur Bispo do Rosário: o senhor do labirinto. Rio de Janeiro: Editora Rocco.

[ref5] Silva JA (1998) Arte e loucura: Arthur Bispo do Rosario. São Paulo: EDUC.

